# The LeVe CPAP System for Oxygen-Efficient CPAP Respiratory Support: Development and Pilot Evaluation

**DOI:** 10.3389/fmedt.2021.715969

**Published:** 2021-08-24

**Authors:** Pete Culmer, W. Davis Birch, I. Waters, A. Keeling, C. Osnes, D. Jones, G. de Boer, R. Hetherington, S. Ashton, M. Latham, T. Beacon, T. Royston, R. Miller, A. Littlejohns, J. Parmar, Tom Lawton, S. Murdoch, D. Brettle, R. Musasizi, G. Nampiina, E. Namulema, N. Kapur

**Affiliations:** ^1^School of Mechanical Engineering, University of Leeds, Leeds, United Kingdom; ^2^School of Dentistry, University of Leeds, Leeds, United Kingdom; ^3^Leeds Teaching Hospitals Trust, Leeds, United Kingdom; ^4^Medical Aid International Ltd., Bedford, United Kingdom; ^5^Bradford Teaching Hospitals National Health Service (NHS) Foundation Trust, Bradford, United Kingdom; ^6^Mengo Hospital, Kampala, Uganda

**Keywords:** CPAP, frugal innovation, respiratory support, medical devices, COVID-19

## Abstract

**Background:** The COVID-19 pandemic, caused by severe acute respiratory syndrome coronavirus 2 (SARS-CoV-2), has placed a significant demand on healthcare providers (HCPs) to provide respiratory support for patients with moderate to severe symptoms. Continuous Positive Airway Pressure (CPAP) non-invasive ventilation can help patients with moderate symptoms to avoid the need for invasive ventilation in intensive care. However, existing CPAP systems can be complex (and thus expensive) or require high levels of oxygen, limiting their use in resource-stretched environments.

**Technical Development** + **Testing:** The LeVe (“Light”) CPAP system was developed using principles of frugal innovation to produce a solution of low complexity and high resource efficiency. The LeVe system exploits the air flow dynamics of electric fan blowers which are inherently suited to delivery of positive pressure at appropriate flow rates for CPAP. Laboratory evaluation demonstrated that performance of the LeVe system was equivalent to other commercially available systems used to deliver CPAP, achieving a 10 cm H_2_O target pressure within 2.4% RMS error and 50–70% FiO_2_ dependent with 10 L/min oxygen from a commercial concentrator.

**Pilot Evaluation:** The LeVe CPAP system was tested to evaluate safety and acceptability in a group of ten healthy volunteers at Mengo Hospital in Kampala, Uganda. The study demonstrated that the system can be used safely without inducing hypoxia or hypercapnia and that its use was well-tolerated by users, with no adverse events reported.

**Conclusions:** To provide respiratory support for the high patient numbers associated with the COVID-19 pandemic, healthcare providers require resource efficient solutions. We have shown that this can be achieved through frugal engineering of a CPAP ventilation system, in a system which is safe for use and well-tolerated in healthy volunteers. This approach may also benefit other respiratory conditions which often go unaddressed in Low and Middle Income Countries (LMICs) for want of context-appropriate technology designed for the limited oxygen resources available.

## Introduction

The COVID-19 pandemic, caused by severe acute respiratory syndrome coronavirus 2 (SARS-CoV-2), has placed a significant demand on healthcare providers (HCPs) to provide respiratory support for patients with moderate to severe symptoms (1). Emerging clinical reports indicate that Continuous Positive Airway Pressure (CPAP) non-invasive ventilation can help patients with moderate symptoms to avoid the need for invasive ventilation in intensive care (2, 3), a change to the first impression that early intubation was indicated. Use of CPAP has been proposed in this context because the positive pressure can help address hypoxaemic respiratory failure, opening the lungs to aid oxygen absorption in lungs which remain compliant and recruitable (4). Regulatory authorities such as the UK Medicines and Healthcare products Regulatory Agency (MHRA) (5), US Food and Drug Administration (FDA) (6) and World Health Organization (WHO) (7) have produced guidance to support rapid development, manufacture and approval of new ventilation systems which can be produced at scale (8–10). However, the demand for ventilator equipment is outstripping supply through complex international supply chains (11). Similarly, the high patient numbers presenting in a clinical setting has placed increased burden on hospital resources and the provision of medical oxygen crucial for ventilation has faced restrictions to avoid overloading hospital systems (12).

**Figure 1:**
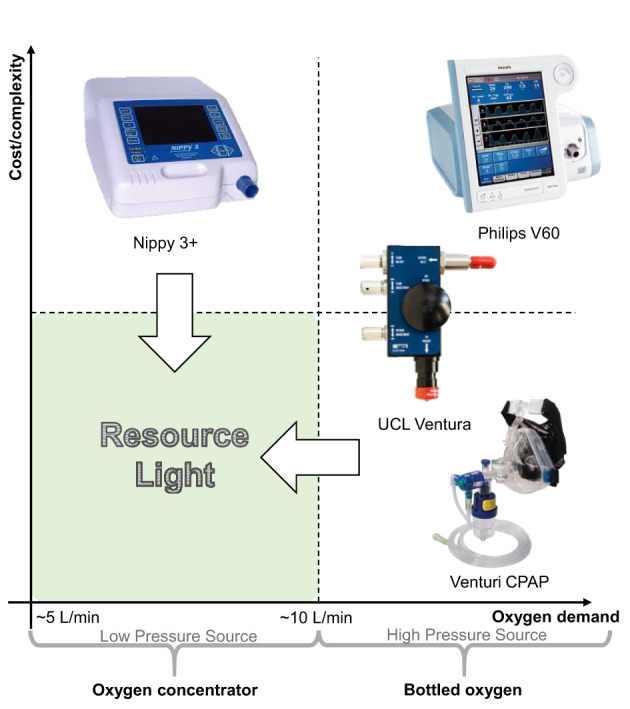
Comparison of non-invasive ventilation options for delivering CPAP arranged into quadrants according to their relative cost/complexity and use of oxygen. The “resource light” quadrant (lower left) highlights the need for systems which are both mechanically simple and can operate sustainably using an oxygen concentrator with typical output flowrates (13).

The need to minimise oxygen consumption per patient and reduce the complexity of equipment are paramount to consider together, this has implications on adoption within different healthcare contexts. Figure 1 uses these traits to classify the types of system available for delivering CPAP in a healthcare setting. The innovation to address provision as a result of the COVID-19 pandemic has been impressive, particularly in relation to systems using pressurised oxygen (the right hand quadrants of Figure 1) typical of many healthcare systems in high income countries (HICs). For example, the UCL-Ventura device (mid-right quadrant) based on a Respironics Whisperflow (14), has been licensed in excess of 1,000 times (9). Whilst the focus of development was on rapid delivery, the final device showed improved oxygen efficiency over its initial design as a result of engineering changes. Venturi valves (bottom right quadrant) are mechanically simple, have been used extensively within healthcare settings to deliver CPAP, and are readily scalable from a manufacturing standpoint. A high pressure source of oxygen flows through the valve and entrains air, creating a flow of enriched air at a modest pressure. Different valve designs give a different FiO_2_ (Fraction of inspired oxygen) which allows clinicians to specify an appropriate valve from a measurement of the patient's oxygen saturation levels, with pressure in the circuit controlled using a Positive End-Expiratory Pressure (PEEP) valve design (15–17). Shortages of these Venturi systems saw groups 3D printing such devices on humanitarian grounds to support patient care (18, 19). However, systems in both the right-hand quadrants require both high flow and high pressure oxygen supply, in part because this provides the energy to generate the pressure and flows within the breathing circuit, rendering them incompatible with oxygen concentrators.

In delivering CPAP, the nature of the oxygen supply in terms of both the flow-per-patient and the delivery pressure is particularly relevant within low-to-middle income countries (LMICs), where limitations in healthcare infrastructure often precludes oxygen delivery from a centralised, pressurised source. Instead, portable oxygen concentrators provide a sustainable, reliable and cost-effective solution (albeit at lower delivery pressures) in contrast to supply from compressed oxygen cylinders which require a reliable supply infrastructure and continual monitoring (13, 20). NGOs such as UNICEF have been provisioning LMICs with oxygen concentrators for many years, recognizing the need for oxygen therapies in general, which is also reflected in their inclusion in the WHO list of essential medicines (21). The oxygen concentrators appropriate for use in LMICs typically output at low pressures and offer flow rates of 5 or 10 L/min with an oxygen concentration of ca. 95% (13). Consequently, to deliver CPAP using oxygen concentrators, systems must fall into the left-hand side of Figure 1. Within the top left quadrant, non-invasive fan based ventilator systems including sleep apnoea systems have been used to successfully treat patients during the COVID-19 pandemic using oxygen entrained near the patient's mask. These have typically focused on treatment of patients with mild to moderate requirements, thus low flow rates of oxygen are used (ca. 5–10 L/min) to obtain 40–60% FiO_2_) (4, 22). This represents the practical lower limit of oxygen consumption since below this, treatment modalities tend to wean patients onto non-enriched CPAP (i.e., no supplementary oxygen is provided) (4). The overall flow rate of the enriched air supplied to the patient needs to exceed at least 20 L/min (to avoid hypercapnia and maintain a positive pressure during inspiration) (23) and in this treatment context is more typically expected to be 60 L/min (24).

However, existing capabilities do not currently address the bottom-left hand corner of Figure 1—we term solutions here as *LeVe* (“light”)—CPAP systems with low complexity and high resource efficiency—both from a design and oxygen supply (flow rate and pressure) perspective. “Frugal innovation” provides a development approach to target this region. Weyrauch and Herstatt (25) define frugal innovation as one where products have: (i) substantial cost reduction; (ii) concentration on core functionalities; and (iii) optimised performance level. Our work targets the development of systems that address the “*resource light*” region, a neglected but important space to consider for Global Health provision. Accordingly, here we report on the development and initial evaluation of the LeVe CPAP Blower, developed using frugal techniques to reduce the complexity of fan based systems and focus *specifically* on providing CPAP functionality.

## System Development

To adapt CPAP technology into a low resource form, such that it is appropriate for use in LMICs as well as other resource-stretched situations, we hypothesise a design through examination of the working principles within existing fan-based non-invasive ventilators.

### Existing Technology

**Figure 2:**
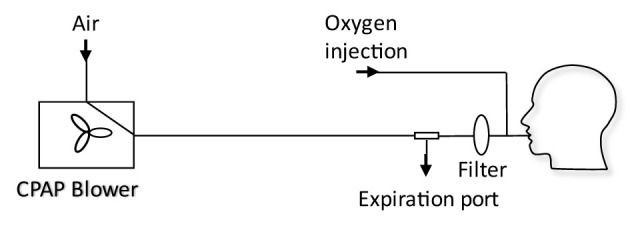
Schematic of a typical CPAP breathing circuit used for COVID-19 treatment (4). The CPAP machine is connected to an expiration port, a HEPA filter, an oxygen inlet port, and the patient mask. The expiration port is a plain hole: filtration of exhaled air before exhaust to atmosphere could prevent aerosolization of disease carrying droplets.

Fan based ventilators use a modulated fan to control the output pressure on a breathing circuit. The desired positive pressure is maintained through a control loop, with an internal pressure sensor used to set the appropriate fan speed. The breathing circuit is shown in Figure 2. Of critical consideration is the need to entrain oxygen close to the patient (between the mask and expiration port) to ensure high oxygen efficiency, while using an expiration port to prevent build-up of CO_2_. An example of a commercial system is the Nippy 3+ (Breas Medical Ltd.), which was adopted successfully within the Leeds Teaching Hospitals NHS Trust to support COVID-19 patients. Such systems have also been demonstrated in an open-source framework with a low raw component cost although the expertise to assemble and guarantee the quality of such a system is still relatively high (25). The functionality of these devices is typically in excess of that required to deliver CPAP therapy alone, systems typically offer more complex breathing support including bi-level positive airway pressure and automatic positive airway pressure. Since adoption into a health care setting is not only about equipment but also training of staff, this additional functionality may be disabled to ensure healthcare compliance.

### Requirements

To inform the development process of the LeVe system, our target requirements were defined as delivery of CPAP at a mean pressure of 10 cm H_2_O (1,000 Pa). The flowrate required to maintain positive pressure is not set *a priori*, but determined to ensure that the pressure remains positive for all parts of the breathing cycle, nevertheless a typical guide value of 60 L/min provided an initial starting point for a suitable flow (24). Within this CPAP regime, the system should achieve a minimum 40% FiO_2_ using an oxygen flow rate of 5 L/min (under normal conditions) supplied at the modest delivery pressures that can be achieved by oxygen concentrators (24); there is some variation across concentrator models, widely used oxygen concentrators such as the Phillips Everflow can be considered representative and have outlet pressures of ca. 38 kPa (387 cm H_2_O) (26). A general observation we have made is that it is not always clear what conditions the flow rates are reported under; when matching low pressure sources with CPAP devices, both the pressure and the flow rate (at this pressure) are required. This allows for total oxygen consumption to be compared (at some defined standard air conditions, e.g., Normal Temperature and Pressure 20°C and 101.325 kPa) as well as establishing the suitability of oxygen sources.

The heterogenous nature of healthcare settings means that adoption of a specific technology requires local assessment against availability of parts, manufacturing facilities and healthcare services. To support this, a wider discussion of overall system requirements is included within this work.

### Technical Approach

The LeVe CPAP Blower system is based on the premise that a category of Brushless DC Current (BLDC) “fan blowers” typically used for thermal management in electrical equipment inherently have the flow dynamics required for provision of CPAP, in general providing relatively high flow rates at low but stable pressures. We propose a design compatible with an oxygen concentrator which intrinsically drives an oxygen efficient solution. Based on frugal engineering principles, the design centers around an appropriately specified, single electric fan-blower without the need for control features of more complex fan-based CPAP systems. The resultant breathing circuit also minimises the number of parts required for effective CPAP since careful choice of the fan and the use of a simple expiration port (which consists of a pipe section with a small hole in the side—typically 4 mm in diameter) means that no PEEP valve (which brings greater complexity and cost) is required.

We examined a range of BLDC fans capable of generating pressures in the range of 12–20 cm H_2_O (1,200–2,000 Pa) at flow rates of ~100 L/min, thus ensuring the flow is greater than the peak inspiratory flow rate to maintain continuous positive pressure through the breathing cycle. By limiting the peak delivery pressure in this selection ensures there is a degree of safety for the patient should any speed regulation fail.

Two multinational manufacturers (selected to help ensure supply-chain availability) supply models which fall within these criteria [*CUI Devices* CBM-979533B-168 (peak pressure 1,325 Pa), *Sanyo Denki San Ace* B97 9BMB (peak pressure 1,280 Pa) and B97 9BMC (peak pressure 1,950 Pa)].

Of these possibilities, we selected the *Sanyo Denki San Ace* 9BMB model because it allows (equivalent) control of the fan speed either by varying the supply voltage or by fixing this and using a Pulse Width Modulation (PWM) control input (the robust 555 timing chip can thus be used). The latter can reduce system complexity and potential failure modes (in comparison to directly varying the fan supply voltage). This model is available in 12 or 24 V models.

**Figure 3:**
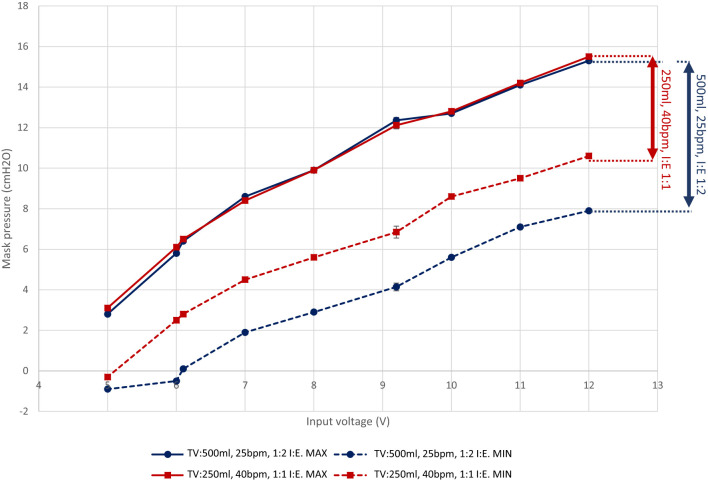
The pressure response of the LeVe Blower with varying supply voltage for different simulated respiratory regimes. Each measurement point represents the mean min/max pressure over 10 breathing cycles (defined as inspiration followed by expiration). Indicative standard error bars for these measures are indicated and range from 0.14 to 0.29 cm H_2_O. Tidal Volumes (TV) of 500 and 250 ml were simulated at 25 breaths per minute (BPM) and different inspiration:expiration (IE) ratios. Connecting lines show the estimated response between measurement points using linear interpolation. Pressures are gauge pressures with sub-ambient pressures recorded within the breathing circuit where inspiration rates are greater than the fan supply.

Figure 3 shows how a fan-blower based system can offer a range of CPAP pressures through modulation of the fan speed (Sanyo Denki San Ace 9BMB12P2K01, here with the speed varied by changing the supply voltage). A breathing simulator, described in Section 4, was used to obtain measures for two representative respiratory regimes. The characteristics show that the voltage input to the fan controls the overall fan speed and consequently the static pressure generated within the system. This offers the ability to adjust the mask pressure by using a voltage regulator to supply a variable supply voltage to the fan. In addition, the minimum pressure differs between the respiratory regimes because they impose different peak inspiratory flow rates (a function of the Tidal Volume and Inspiration:Expiration ratio). The fan cannot respond instantaneously to changes in flow rate and consequently a pressure drop occurs. The inspiratory flow rate is higher for the 500 ml TV regime in comparison to the 250 ml TV regime and consequently the minimum pressure is lower. A working range can be defined between the maximum operating speed and the point at which the fan cannot meet the required flow rates. In this instance, below 7 V the mask pressure for the 500 ml respiratory regime is negative; under such a situation the instantaneous peak inspiratory flow rate is higher than the maximum flow supplied by the fan, so a positive pressure cannot be maintained (shown as a negative absolute pressure in Figure 3).

### System Design

**Figure 4:**
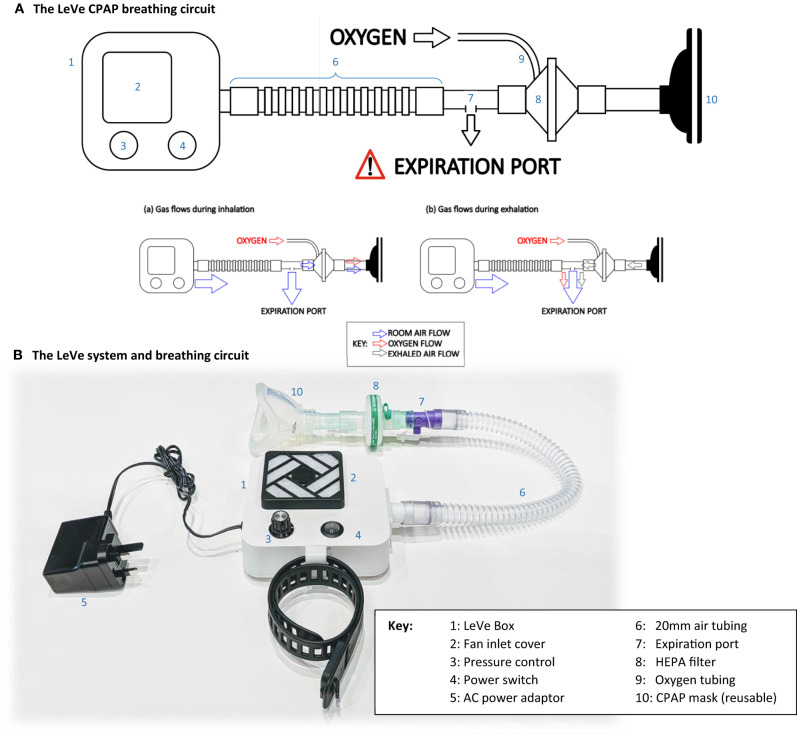
**(A)** The LeVe CPAP breathing circuit and **(B)** The complete LeVe system.

The complete LeVe CPAP system was developed after selection of the blower-fan to provide a robust package appropriate for use in low-resource contexts by trained medical practitioners. The system is designed around the fan blower, combined with a standard breathing circuit, in the configuration shown in Figure 4. In this design, rather than controlling through a supply voltage, the fan speed is controlled through a pulse-width modulated signal, generated using robust 555 timing chips on a low-cost PCB, which provides a low-voltage control frequency directly to the fan. A four-way dial allows selection of nominal CPAP pressures of 5, 7.5, 10, and 12.5 cm H_2_O, selected to match typical requirements in COVID-19 treatment and with an upper limit advised by our clinical team (27). Power is provided to the selected fan (9BMB24P2K01) through a medical grade 24 V powerpack, which accepts a wide input voltage of 80–264 V AC. We note that a reduced part count could be achieved by connecting the fan directly to 24 V (for this fan model) to give a fixed CPAP pressure of 12.5 cm H_2_O. A single switch on the DC circuit allows the unit to be turned on and off. An intake filtre is provided to prevent particulate material entering the fan unit. The LeVe CPAP breathing circuit integrates the LeVe flow generator, to give an oxygen efficient breathing circuit, as shown Figure 4A. The breathing circuit has a dead space of ~320 ml (comprising the volume of the mask and breathing circuit up to the expiratory port). The breathing circuit was implemented under the guidance of the clinical team within the Leeds NHS Teaching Hospital Trust and Bradford NHS Teaching Hospital Trust for treatment of patients with Covid-19 and adopted nationally within the UK (4, 28).

From the LeVe flow generator, there will be a constant flow of air through the expiration port. During inspiration, a fraction of air is drawn from this flow toward the patient and this oxygen is entrained into this fraction. This mechanism ensures enrichment of just the air breathed by the patient rather than requiring the entire airflow to be enriched at source. Exhaled air passes out of the exhalation port, together with some oxygen, which represents a small inefficiency within the circuit. However, the overall oxygen efficiency is still high, particularly compared to systems like Venturi devices which typically use over 15 L/min oxygen from a high pressure (3–4 bar) mains source to achieve a working pressure of 10 cm H_2_O (24, 29). The ultimate FiO_2_ is then be controlled by varying the flow rate of the oxygen source appropriately. It is important to note that this is intended to be performed by a trained clinician informed by oxygen saturation readings [as performed for similar CPAP systems in clinical use (4)].

## System Evaluation

The fundamental performance characteristics of a CPAP system can be measured through: (i) the pressure at the patient mask during a breathing cycle; and (ii) the FiO_2_ of inspired air for a given oxygen flow rate. These aspects were investigated in controlled laboratory conditions to ensure appropriate performance prior to human use.

### Methods

**Figure 5:**
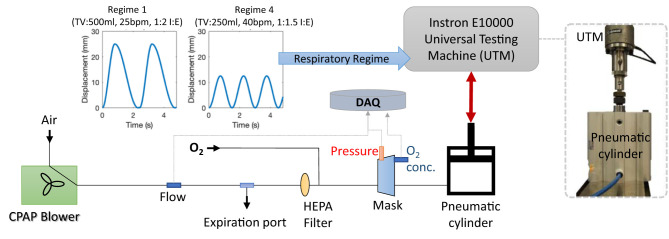
The breathing model and test configuration used to evaluate the LeVe systems. The pneumatic cylinder can be driven by the Universal Testing Machine to simulate different respiratory regimes, as defined by Tidal Volume (TV), Breaths per Minute (BPM) and the Inspiration:Expiration ratio (I:E). All data were logged to the data acquisition system (DAQ).

To measure the flow dynamics of the system in a controlled and repeatable manner, a breathing simulator was developed. The simulator, summarised in Figure 5, consisted of a large bore pneumatic cylinder (SMC CQ2 Series, 160 mm diameter) driven by a dynamic testing machine (Electro Puls E10000, Instron) which allows the cylinder piston to be moved under a pre-defined cyclic pattern, facilitating a variety of respiratory regimes (see Figure 5, inset, for example) to be tested. The rigid cylinder removes the effect of lung compliance on the simulations but enables the impact of breathing patterns to be assessed in terms of oxygen efficiency and pressure response of the circuit. Four different respiratory regimes were used (Table 1) to represent a spread of respiratory cases, from slow deep breathing in healthy adults to more rapid shallow breathing associated with conditions like COVID-19 (33).

**Table 1:** Respiratory regimes used to evaluate the LeVe system defined by the simulated lung parameters Tidal Volume (TV), Breaths per Minute (BPM) and Inspiration:Expiration (I:E) ratio (the time taken for each phase of the breathing cycle).

**Respiratory regime**	**TV (ml)**	**BPM**	**I:E ratio**	**Reference**
(1) (Baseline)	500	25	1:2	Brusasco (24)
(2) (Modified baseline)	500	25	1:1.5	–
(3)	500	20	1:1.5	Schneider, Wilkins (30, 31), UK MHRA (5)
(4)	250	40	1:1	Kallet et al. (32)

Key parameters measured within the system were mask pressure (IPSU-M12, RS), outlet flowrate of the blower (SFM3300, Sensiron), and oxygen concentration in the mask (Max-550E, Maxtec). Pressure and flowrate data were logged using a data acquisition system (cRIO, National Instruments) at 200 Hz whilst the oxygen concentration was recorded once a steady state was reached. In each configuration, measurements were averaged over 10 cycles. Oxygen was supplied using a concentrator (Drive 10 L/min DeVilbiss Healthcare). Each system was connected to the breathing simulator using standard medical-grade components.

### Results

**Figure 6:**
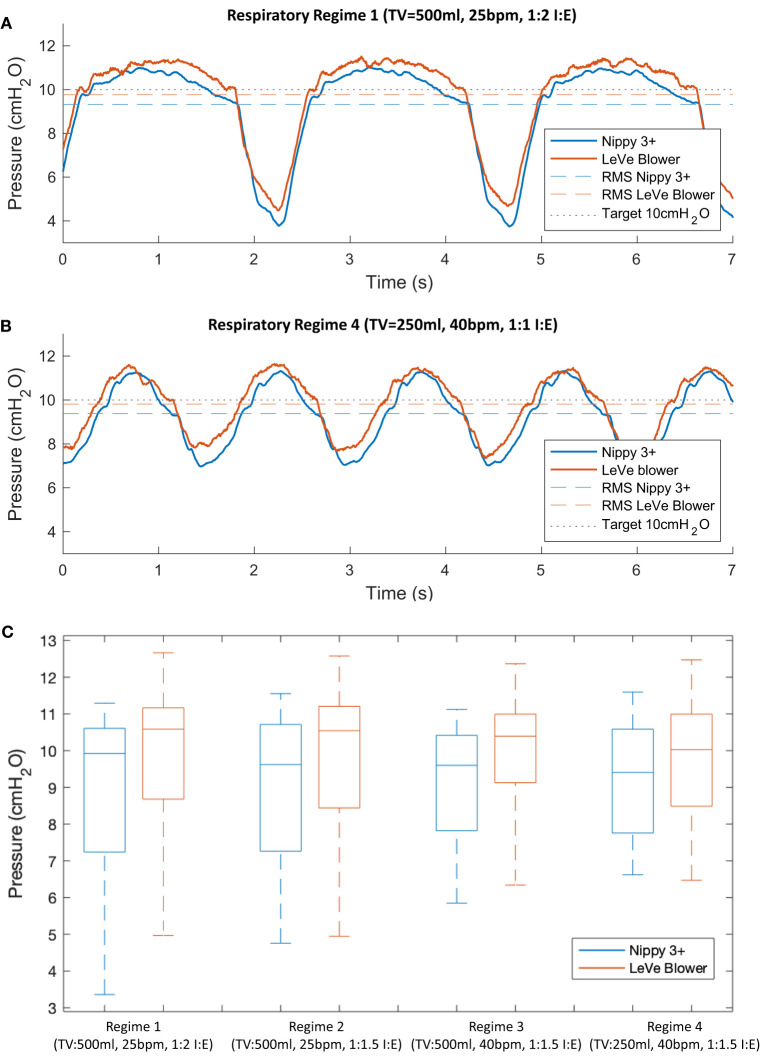
Typical pressure response characteristics of the LeVe system and a commercial sleep apnea CPAP system (Nippy 3+), configured to generate 10 cm H_2_O (Indicated by black dotted line) shown for different respiratory regimes, defined by Tidal Volume (TV), Breaths per Minute (BPM), and Inspiration:Expiration ratio (I:E). Parts **(A,B)** show representative pressure waveforms for two contrasting respiratory regimes, **(C)** provides a boxplot summary of the pressure characteristics across all respiratory regimes defined in Table 1.

Figure 6 shows a comparison of the mask pressure achieved for two respiratory regimes with the LeVe system and the commercial Nippy 3+, both configured to provide 10 cm H_2_O. In general, a drop in pressure is observed during inspiration as air is drawn from the breathing limb, and a rise observed during expiration. For the baseline respiratory regime 1 (see Table 1) the RMS pressure of the LeVe system is 9.76 cm H_2_O (2.4% error), in comparison the Nippy 3+ achieves 9.32 cm H_2_O (6.8% error). Across the breathing cycle the pressure range of the LeVe is 7.04 cm H_2_O and for the Nippy 3+ is 7.29 cm H_2_O. These characteristics are similar to those recorded for other commercial and open source CPAP systems (34).

Figure 6C presents a summary box plot indicating the median, upper, and lower quartile as well as the maximum and minimum values. Unsurprisingly, given the observations in Figures 6A,B, the pressure characteristics for the two systems are comparable with respect to their median pressure and overall operating range. The pressure range is affected by the respiratory regime since this defines the peak inspiration flow rates. For example, the LeVe system produced a pressure range of 7.04 cm H_2_O (Regime 1) and 4.32 cm H_2_O (Regime 4). It should be noted that pressures remained positive in all cases, which is important to ensure the lungs remain open.

**Figure 7:**
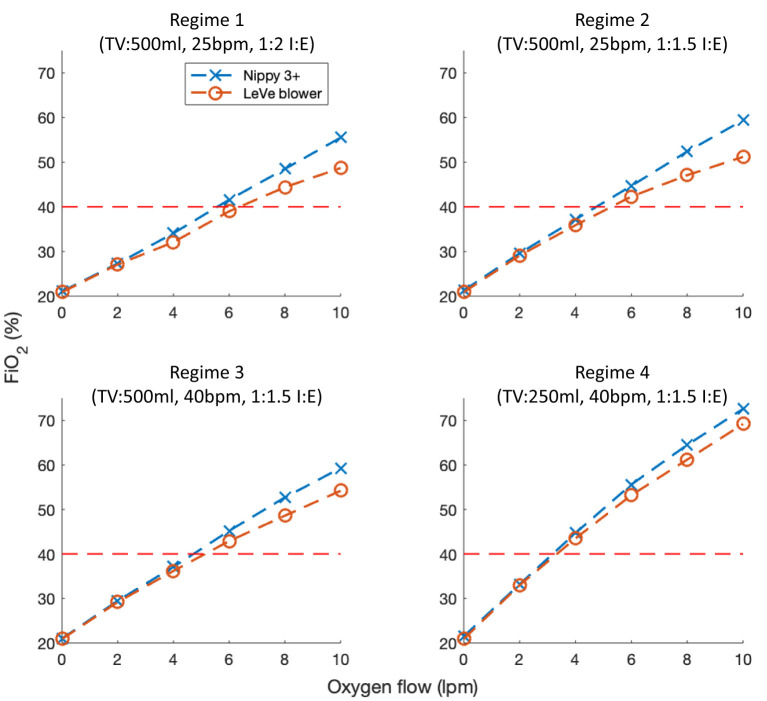
FiO_2_ characteristics of the LeVe breathing circuit in comparison to a sleep apnea CPAP system (Nippy 3+) under different respiratory regimes defined by Tidal Volume (TV), Breaths per Minute (BPM), and Inspiration:Expiration ratio (I:E). Oxygen flow rates reported under NTP.

Figure 7 illustrates the oxygen efficiency of the LeVe system alongside that of the Nippy 3+. In terms of oxygen performance, the Nippy 3+ was found to have a slightly higher efficiency (i.e., the FiO_2_ achieved for a given oxygen supply flowrate) than the LeVe system. More significantly, the respiratory regime has an effect on oxygen performance, in particular lower peak inspiration rates (lower breaths per minute, smaller tidal volumes, and/or lower I:E ratios) resulting in increased efficiency and a reduction in the difference between systems. In both systems, the maximum FiO_2_ that can be achieved at 10 L/min O_2_ (i.e., the limit of a typical oxygen concentrator) ranges from ca. 50 to 55% (TV = 500 ml, 25 bpm, I:E 1:2) to 70% (TV = 250 ml, 40 bpm, I:E 1:1.5).

## Pilot Study

A pilot study was conducted to evaluate the safety and acceptability of the LeVe CPAP Flow Generator in a group of healthy volunteers at Mengo Hospital in Kampala, Uganda. Two research questions were set:

Primary: Can the LeVe CPAP Flow Generator be used safely without inducing hypoxia or hypercapnia? Secondary: Is the LeVe CPAP Flow Generator well-tolerated by users?

The healthy participants in the study are likely to present with lungs with normal lung compliance. While this does not necessarily replicate the lung condition of those with COVID-19 (the motivation for this work) it is a necessary first stage in demonstrating that the system can provide a safe and tolerable intervention.

### Methods

This study took place in the Intensive Care Unit at Mengo Hospital, Kampala, Uganda. A sample of 10 participants was recruited, all of whom were members of staff at the hospital. All participants were provided with a written information sheet, gave informed written consent and were not offered any financial incentive to participate in the study. Approval was obtained from the Mengo Hospital Research and Ethics Committee (M 02/01-2021) and the Uganda National Council for Science and Technology (HS1250ES). The protocol was registered and approved by the Pan African Clinical Trials Registry (PACTR202105734146484), a summary of key aspects is as follows. The inclusion criteria were for participants to be staff members at Mengo Hospital aged 20–50 years with no involvement in the research study. The key exclusion criteria were:

- Current or ex-smoker
- Underlying respiratory conditions
- BMI > 30
- Any contraindications from previous use of CPAP or oxygen therapy.

The participants first trialled the mask to assess the fit. After taking a baseline reading, the LeVe CPAP Flow Generator was switched on and the pressure was stepped through the 4 pressure settings (5, 7.5, 10, and 12.5 cm H_2_O). A commercially available oxygen concentrator was connected to the breathing circuit (as shown in Figure 4) to enrich the air supply with 5 L/min oxygen. The participants' oxygen saturation (SpO_2_) and end tidal CO_2_ (ETCO_2_) level were monitored continuously throughout the study. Oxygen saturation was measured by a pulse oximeter (Mindray VS900) with finger attachment, ETCO_2_ using a capnograph with the sample line attached to a port on the face mask. Hypoxia was defined as oxygen saturations <94% and hypercapnia was defined as ETCO_2_ > 5.7 kPa (35). The null-hypothesis was no difference in oxygen saturation across the five groups represented by the four CPAP values and the baseline measurement. A one-way ANOVA was undertaken with a *post-hoc* assessment of pairwise evaluations using the Bonferroni Correction to account for multiple comparisons. The participants were then provided with a questionnaire which asked them to rate overall comfort, anxiety, claustrophobia, and difficulty in breathing using a Likert scale to assess each attribute. This was adapted from a similar study (36) and is available in Supplementary Materials.

### Results

In total, 10 participants were recruited. Mean age was 24.9 years (range 22–30 years) and 50% were female. Measured data for end-tidal CO_2_ and oxygen saturation levels is provided in Table 2. Data were recorded successfully for all participants with the exception of two readings at 5 cm H_2_O for participants 9 and 10 due to a reading error at this setting. Overall, the results demonstrate a consistent and desirable positive response in oxygen saturation levels of 96–100% SpO_2_ (37) across all participants, within accepted healthy limits. Similarly, end tidal CO_2_ ranges between 3.6 and 4.9 kPa across the participants, all below the 5.7 kPa threshold that was defined as hypercapnia (35).

**Table 2:** Oxygen saturation and end-tidal CO_2_ levels across the cohort (MD, Missing data due to reading error).

**Participant**	**% Oxygen saturation**					**Maximum ETCO_**2**_ (kPa)**
	**CPAP setting (cm H**_**2**_ **O)**					
	**0**	**5**	**7.5**	**10**	**12**	
1	97	98	98	98	98	3.6
2	97	98	98	98	98	4.8
3	98	98	98	99	100	4.6
4	98	98	98	98	98	4.3
5	97	98	99	99	99	4.8
6	96	95	98	98	98	4.6
7	96	98	98	98	98	3.9
8	96	96	98	98	99	3.9
9	97	MD	98	99	99	4.5
10	97	MD	96	98	98	4.9
Mean	96.9	97.4	97.9	98.3	98.5	4.4

**Figure 8:**
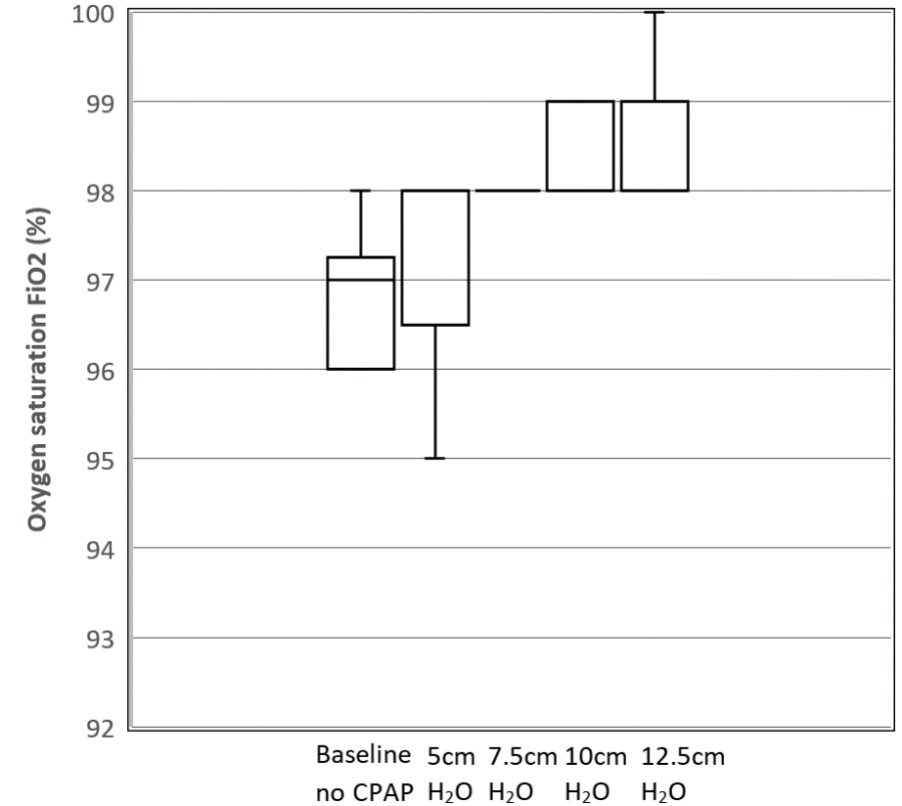
Box and whisker plot showing mean oxygen saturation levels as a function of CPAP levels for the 10 participants. *N* = 10 for Baseline, 7.5, 10, 12.5 cm H_2_O; *N* = 8 for 5 cm H_2_O (due to missing data).

Figure 8 shows the average oxygen saturation levels for the participants as a function of the nominal CPAP setting. The ANOVA revealed a rejection of the null-hypothesis (*P* = 0.0002) across the five groups with significant pairwise comparisons noted for both groups as denoted by CPAP values of 10 and 12 cm H_2_O when compared with the base readings in the absence of CPAP. This indicates a small but detectable improvement in oxygen saturation in participants with healthy lungs at the two highest values of CPAP pressure, potentially because this acts to reduce the physiological shunt. However, this outcome requires further study in a non-healthy population before any clinical significance can be attributed.

**Figure 9:**
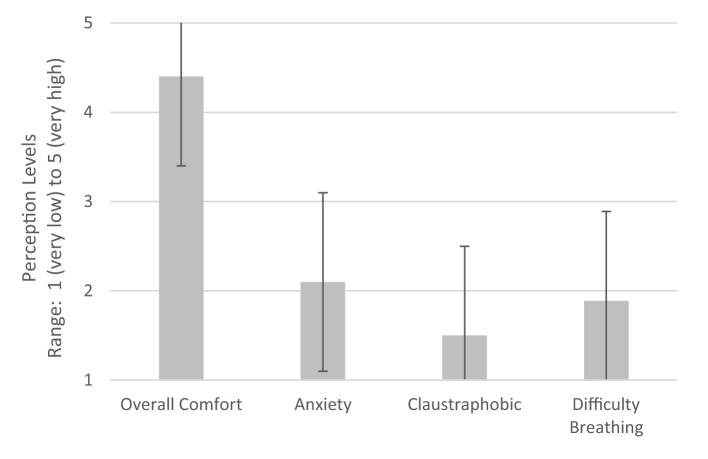
User tolerability of device. Perceptions of users during CPAP delivery using LeVe. Error bars indicate ±1 SD. 5: Very high, 4: High, 3: Moderate, 2: Low, and 1: Very low.

In terms of tolerability, overall comfort was measured on a scale of 1–5 where 1 indicated “not at all comfortable” and 5 indicated “very comfortable.” Figure 9 demonstrates user tolerability of the device with the mean response and standard error also shown. Mean overall comfort level was 4.4. Anxiety, claustrophobia, and difficulty in breathing were then measured on a scale of 1–5 where 1 indicated “not at all” and 5 indicated “strongly.” Mean anxiety level was 2.1, mean claustrophobia level was 1.5, and mean difficulty in breathing level was 1.9.

## Discussion

The LeVe Blower is a simple system, deliberately developed to provide a low resource solution for the provision of CPAP ventilation in terms of oxygen requirements, power, and ease-of use whilst not sacrificing performance. Following frugal engineering principles, the systems have been developed to meet a focussed set of requirements while removing extraneous functionality and thus complexity. For example, while open-sourced CPAP systems have been developed to provide low-cost alternatives to commercial systems, they fundamentally share a common approach in using microprocessor systems to regulate their output (34). In contrast, the LeVe system uses the inherent flow characteristics of the fan to achieve comparable performance in terms of RMS pressure (e.g., 10 cm H_2_O target pressure within 2.4% RMS error) and 50–70% FiO_2_ over representative respiratory regimes, despite their lack of closed-loop pressure regulation. This has a small impact on oxygen performance (shown in Figure 7) because the open-loop fan control reduces the fraction of exhaled air that is rebreathed and that which leaves the system, hence it achieves marginally lower FiO_2_ for a given oxygen supply flowrate. Considering resource efficiency, the LeVe system is designed to generate a pressurised airflow without the use of compressed oxygen, which is typically a limited commodity in LMICs, instead relying on more prevalent and sustainable electrical power (13, 26). This approach enables the system to achieve high efficiency in the supply of oxygen-enriched air and allows oxygen delivery to be controlled independently of the desired CPAP operating pressure. In the case of LMIC context, this enables the use of oxygen concentrators as a supply.

Selection of an appropriate CPAP system for clinical use is heavily dependent on the environment, infrastructure, and resources present. Reflecting on Figure 1, our focus has been to target “resource-light” solutions, to ensure that they are appropriate for LMIC contexts. This goes beyond producing a “low-cost” system, instead frugal engineering emphasises the need for systems which consider manufacture, sustainable long-term use and crucially does not sacrifice performance to achieve these goals. The LeVe system is designed to operate using an oxygen concentrator to enrich the air supply. Typically, these are available in 5 or 10 L/min where the output of the latter can be split with 2 flow regulators to treat two patients to achieve ~40% FiO_2_. This provides a scalable and resource-efficient solution to cater for varying patient numbers. In contrast, operating conventional Venturi valve systems, which fall in the lower right quadrant of Figure 1 (and therefore have a cost/complexity profile appropriate for LMIC), would require provision of compressed oxygen at high flow rates which is challenging to achieve in many LMICs without recourse to repeated changeover of oxygen cylinders, a practise which is both costly and demands regular maintenance support (38).

The results of the study demonstrate that in a healthy cohort, the LeVe system is safe for use and well-tolerated by the participants. Measures of oxygen saturation demonstrate that the system does not induce hypoxia or reduce oxygen saturation, and in fact it may actually increase saturation levels. In a fit and well-population, the significance of any increase in SpO_2_ with CPAP was likely to be minimal. Similarly, measures of end tidal CO_2_ show that LeVe does not cause hypercapnia. The ultimate FiO_2_ delivered by any CPAP system varies with respiratory function and is not explicitly controlled. Thus, these systems require external monitoring by a suitably qualified healthcare professional based upon the patient's SpO_2_ level and vital signs in accordance with best practise (e.g., UK MHRA guidance). This places an emphasis on the need to accompany such systems with appropriate training and clinical use protocols to ensure quality of care.

Considering comfort, it is important to note that the provision of positive pressure through a face mask will inherently impact on “natural” inhalation and expiration, thus may cause anxiety and discomfort, regardless of the air source (39). Within the scope of this study, it indicates that the air pressure and flow characteristics of the LeVe system, in particular the inherent variability which occurs during the breathing cycle, are both tolerated and appropriate for future clinical evaluation. In conjunction, the high comfort rating reported for the mask is integral to the overall experience and should be carefully selected to ensure a close but comfortable fit.

Research has shown how the clinical efficacy of CPAP systems has evolved rapidly during the COVID-19 pandemic (3). Particularly in LMIC contexts, the utility of CPAP is likely to have increasing relevance to treatment of other conditions, particularly when more advanced forms of ventilation are not available. For example, CPAP provides a route to stabilise patients with acute pulmonary oedema whilst the underlying cause is being treated (40). Similarly, based on the success of using CPAP to treat COVID-19, it could be explored for treatment of conditions like viral pneumonias or severe influenza. This need not be confined to acute settings, there is scope to explore the use of simple devices like LeVe for early presentation of COVID-19 within community settings, helping to reduce the burden on hospital admissions. Last, but not least, it is also interesting to note that while these systems were developed to target use in LMICs, there is increasing recognition of the need to innovate for value within the resource-strained healthcare systems of HICs. Often termed “reverse innovation,” there is also potential for the use of low-resource CPAP systems within services like the UK's NHS (41, 42).

## Conclusions

CPAP ventilation systems provide an important treatment option for COVID-19 patients, particularly in the early stages before invasive ventilation strategies are required, to deliver oxygen-enriched air to stabilise patients until they can be escalated or de-escalated. To deliver this for the high patient numbers associated with the COVID-19 pandemic, healthcare providers require resource efficient solutions. We have shown that this can be achieved through frugal engineering of a CPAP ventilation system.

The data from the pilot study indicate that the LeVe CPAP Flow Generator is safe to use in healthy volunteers and was well-tolerated by the cohort. This solution has different merits in clinical performance and efficiency to existing CPAP systems but provides resource-limited healthcare providers with a more resource-efficient solution to support flexible treatment pathways that can be rapidly deployed to reduce the burden on ICU during the COVID-19 pandemic. Beyond this immediate need, there is also evidence that CPAP can help provide much needed therapeutic benefit to address other respiratory conditions (e.g., respiratory distress syndrome) which often go unaddressed in LMICs for want of context-appropriate technology.

Our ambition is that this work will support the treatment of patients suffering from COVID-19 and (beyond the current pandemic) expand treatment options available to healthcare professionals targeting respiratory distress syndromes. Our ongoing work will build on these foundations; firstly by evaluating the clinical efficacy of using the LeVe system to provide respiratory support in patients with COVID-19, secondly by exploring opportunities to make the technology commercially available, coupled with the requisite regulatory approval, for use in LMIC settings.

## Data Availability Statement

The original contributions presented in the study are included in the article/Supplementary Material. Further information on the LeVe system is available through license at: https://licensing.leeds.ac.uk/.

## Ethics Statement

The studies involving human participants were reviewed and approved by Mengo Hospital (Kampala, Uganda) IRB and the Uganda National Council for Science and Technology approved this study. The patients/participants provided their written informed consent to participate in this study.

## Author Contributions

PC and NK supervised the project and wrote the manuscript. WD and IW manufactured, tested, and analysed results from the systems. DJ, RH, AK, and CO provided technical assistance in the system development. GB, SA, ML, TB, and TR provided technical information to inform development of the system. TL, JP, RMi, and SM provided clinical expertise to guide development and evaluation. DB coordinated independent evaluation of the system. RMu, GN, AL coordinated and conducted the safety study at Mengo Hospital. EN was principal clinical investigator, supervising the safety study at Mengo Hospital. All authors contributed to the article and approved the submitted version.

## Conflict of Interest

TR and RMi were employed by company Medical Aid International Ltd. The remaining authors declare that the research was conducted in the absence of any commercial or financial relationships that could be construed as a potential conflict of interest.

## Publisher's Note

All claims expressed in this article are solely those of the authors and do not necessarily represent those of their affiliated organizations, or those of the publisher, the editors and the reviewers. Any product that may be evaluated in this article, or claim that may be made by its manufacturer, is not guaranteed or endorsed by the publisher.

## Supplementary Material

Supplementary material contains technical documentation on fan selection and system performance.
